# Retrospective Imaging Study of Submandibular Space Infection

**DOI:** 10.7759/cureus.114668

**Published:** 2026-08-17

**Authors:** Hashim G Elmshiti, Aimen Hassan, Luciano Prevedello, Enas H Hassan, Hamid Nurrohman, Gregory Ness, Jahanzeb Chaudhry, Kirk Mchugh

**Affiliations:** 1 Biomedical Science and Dental Science, Missouri School of Dentistry and Oral Health (MOSDOH), A.T. Still University (ATSU), Kirksville, USA; 2 Information Technology, Safelite Group Inc., Columbus, USA; 3 Radiology, The Ohio State University College of Medicine, Columbus, USA; 4 Anatomy, Chamberlain University, Columbus, USA; 5 Restorative Dentistry, The University of Texas School of Dentistry, Houston, USA; 6 Oral and Maxillofacial Surgery, College of Dentistry, The Ohio State University, Columbus, USA; 7 Oral and Maxillofacial Radiology, Hamdan Bin Mohammed College of Dental Medicine, Mohammed Bin Rashid University of Medicine and Health Sciences (MBRU), Dubai, ARE; 8 Anatomy, College of Medicine, The Ohio State University, Columbus, USA

**Keywords:** clinical variables, computed tomography images, facial space infection, systemic conditions, treatment outcomes

## Abstract

The purpose of this study was to determine the clinical variables, including demographic characteristics, comorbidities, and anatomical extent, associated with the severity of submandibular space infections and their treatment outcomes. A retrospective analysis was performed on computed tomography scans of patients diagnosed with submandibular space infections. Demographic characteristics, comorbidities, anatomical extent of the infection, and treatment details were obtained from electronic health records. The primary outcomes were the degree of infection and the results of the treatment, including hospital length of stay. The existence of complications, such as obstruction of the airway or the need for repeated surgical procedures, was also evaluated. Outcomes were analyzed using χ^2^ tests and independent-samples *t* tests. Records of 103 patients were reviewed. The mean (standard deviation (SD)) age was 39.3 (12.6) years and ranged from 18 to 73 years. Men (52, 50.5%) and women (51, 49.5%) were almost equally affected by infection. For all patients, hypertension (18, 17.4%) and diabetes (11, 10.6%) were the most prevalent systemic conditions. Most patients (93, 90%) had anatomical spread to adjacent facial spaces. Twenty patients (19.4%) had airway narrowing as a complication, which was associated with a longer hospital length of stay (*P* = 0.04). Infection in the retromolar trigone and submental spaces resulted in repeated surgical procedures (both *P *< 0.04). In the current study, spreading of infection to the retromolar and submental facial spaces was identified as the variable most strongly associated with increased extent of infection. These results emphasized the importance of early identification and treatment of submandibular space infections and highlighted clinical variables that may impact the infection severity and treatment effectiveness.

## Introduction

Maxillofacial infection is one of the most common sources of head and neck infections and may be life-threatening if it spreads to the deep facial spaces associated with vital structures [1]. These infections generally involve the salivary glands, epiglottis, tonsils, or retropharynx. Further, they can break down the barriers between the fascial spaces formed by the deep cervical fascia [2]. Dentoalveolar abscesses are the most common cause of these deep fascial infections. Although antibiotics can reduce the risk of odontogenic infections, the spread of infection into the deep spaces of the head and neck may eventually involve the mediastinum, pleuropulmonary spaces, and heart valves, leading to severe or fatal outcomes [3].

The diagnosis and surgical treatment of maxillofacial infections are clinically complicated [4], so accurate diagnosis is vitally important. Advanced imaging techniques, such as computed tomography (CT), have been used to assess and treat these infections. Because odontogenic infections spread quickly through the various fascial planes, successful surgical treatment depends on early diagnosis. In a study by Smith et al. [5], there was a positive relationship between obtaining a CT image with contrast-enhanced material and surgical outcome, resulting in improved treatment effectiveness and better clinical outcomes.

Because odontogenic infections are unpredictable, more studies are needed to evaluate the spread of the infection and the associated complications related to treatment, such as hospital length of stay (LOS). From the patient’s perspective, hospital costs and LOS are important factors when a surgical intervention is recommended. However, few studies have investigated the factors contributing to LOS variations [6]. Therefore, the purpose of the current retrospective study was to determine the clinical variables, including demographic characteristics, comorbidities, and anatomical extent, associated with submandibular space infections and their treatment outcomes. More specifically, we used CT scans to evaluate the potentially serious consequences of the spread of submandibular space infection to adjacent head and neck spaces and to assess the potential associations of these infections with serious complications.

## Materials and methods

This study used a retrospective design to evaluate clinical variables associated with the extent of submandibular space infections and their treatment outcomes. The reviewed medical charts were obtained from the Electronic Health Record and Information Warehouse of the Ohio State University Wexner Medical Center. We provided the repository staff with International Classification of Diseases-9 (ICD-9) diagnosis codes for odontogenic infections of the head and neck and Current Procedural Terminology (CPT) codes for the corresponding office procedures, head and neck surgery, and radiology for those diagnoses. To be included in the review, patients had to be clinically diagnosed with submandibular infection with or without involvement of other fascial spaces between November 2011 and September 2014. Any case without submandibular space infection was excluded. Only adult patients were included in the study, so records of patients younger than 18 years and older than 73 years were also excluded. Data from the included records were extracted based on diagnosis and type of surgery. The resulting data were de-identified by a biomedical informatics data analyst and then sent to study researchers. Data were provided for the following variables: demographic characteristics, laboratory results, and CT images. All patients underwent contrast-enhanced axial computed tomography at 3-mm thickness. All images were obtained using the helical technique after intravenous administration of a nonionic contrast-enhanced material and scanned from the top of the aortic arch through the skull base. Using this radiopaque material, the CT images showed a maxillofacial infection as a low-attenuation area surrounded by an enhancement rim of varying degrees of density. The lesion could appear lobulated, multifocal, or elongated. We also obtained a clinical report for each patient that included the extent of infection, etiology of the infection, time of hospital admission, time of operation, and time of hospital discharge. The local institutional review board approved the current study, and no identifying information was provided for the reviewed patient records. Because of the retrospective and anonymous nature of the study, we did not obtain informed consent from the patients whose records we reviewed.

Study outcomes

The primary study variables were demographic characteristics, comorbidities, extent of infection (classified according to the severity of the clinical presentation and extent of involvement), anatomical extent of the infection, and treatment details. Our primary outcomes were the degree of infection, hospital LOS, and treatment outcomes. The existence of complications, such as obstruction of the airway or the need for repeated surgical procedures (second operation), was also evaluated.

The demographic characteristics included in the current study were age, sex, and race. Race was included in the current study because it is a standard demographic characteristic included in medical charts. Because predisposing factors can contribute to the severity of infection and a longer recovery process [6], the following comorbidities were included: hypertension, diabetes, renal disease, and advanced-stage malignancy. Since the third mandibular molar is usually the cause of submandibular space infections [7,8], we only included patient records where the extent of infection was caused by dental caries, periodontal disease, or trauma. We also included anatomical extent as a variable because understanding these features may explain how an infection spreads and identify involved structures. The specific anatomical spaces included in the study were the following: the buccal space, sublingual space, submental space, masticator space, parotid space, parapharyngeal space, carotid space, retropharyngeal space, and mediastinum. Infection in these anatomical spaces was categorized as multiple space infections, which involved submandibular space infection and infections in multiple other anatomical spaces, or as single space infections, which involved submandibular space infection and infection in a single anatomical space.

To evaluate the extent of infection, we categorized the extent as low, moderate, or high [9]. Categories were based on the proximity of the infection to the airway and other vital structures, for example, the heart [9]. Hospital LOS was calculated by using the time interval in calendar days between the admission and discharge times from the medical record.

**Figure 1:**
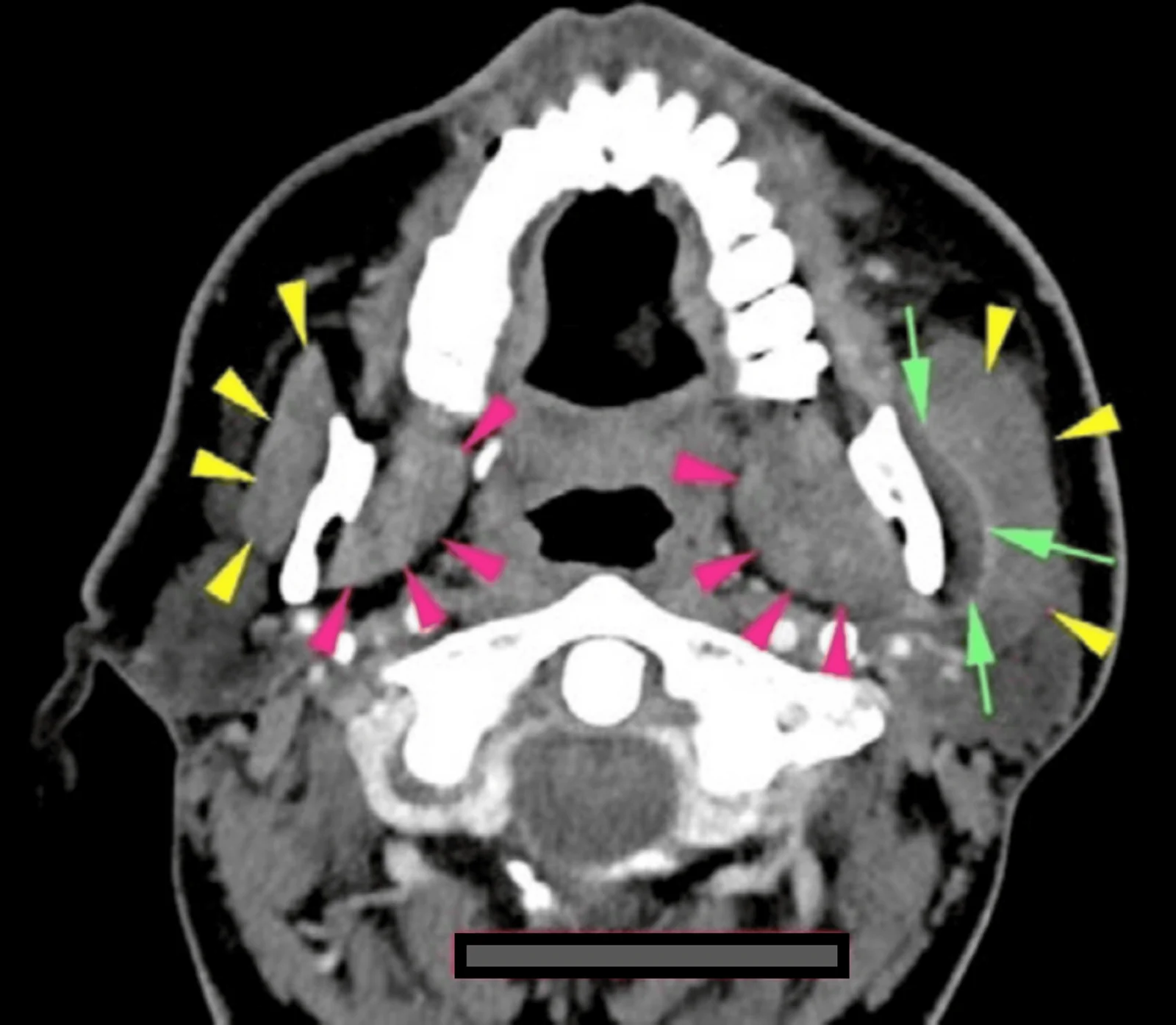
Axial CT reveals a masticator space abscess. The left masseter muscle is enlarged and edematous (yellow arrowheads). A rim-enhancing fluid collection is visible in the left masseter (green arrows). The left medial pterygoid muscle is enlarged and edematous (red arrowheads).

The CT scans were evaluated for the presence of an abscess or cellulitis. An abscess is a cavity with purulent exudate fluid and necrotic tissues. It can be drained surgically and is visible in CT images (Figure 1) as a fluid collection surrounded by a rim of enhancement. Cellulitis is a diffuse inflammation of soft tissues without purulent cavities and cannot be drained surgically. It is visible in CT images as an attenuation area without a surrounding rim of enhancement [4]. Outcomes from a review of the CT scans were characterized as an abscess, cellulitis, bacterial gas, enlargement of the submandibular gland, airway narrowing, trismus, or enlargement of the parotid gland. We also recorded the side of infection (right or left).

Because all patients in this study had surgical incisions and drainage of infected tissues, we assessed surgical complications. Complications included in the study were difficulty with breathing, endotracheal intubation, airway obstruction, trismus, second surgery, and enlargement of the muscles and glands adjacent to the infection.

Statistical analysis

Study variables from patient charts, including demographic characteristics, comorbidities, and anatomic extent (spread of infection) into the different facial spaces, were summarized using frequency and percentage or mean and standard deviation (SD). To evaluate the effect of age on infection, we compared the age of patients by range (18-20, 21-30, 31-40, 41-50, 51-60, and 61-70 years) with facial infection. For subsequent analyses of our data, we defined a second operation and LOS as outcome or dependent variables and the study variables from patient charts as predictive or independent variables. Paired and independent sample t-tests were used to compare hospital LOS with surgical complications. A χ2 test was used to compare those who had a second operation with the anatomic extent of the infection. As a sensitivity analysis, a Mann-Whitney U test was performed for the Airway narrowing comparison because the normality assumption for the t-test was violated. An additional sensitivity analysis was conducted excluding one extreme LOS value to assess its influence on the results. All analyses were conducted using SPSS, version 22 (IBM Corp., Armonk, NY), and a *P*-value ≤ 0.05 was considered statistically significant. This study was conducted in accordance with the STROBE guidelines.

## Results

This retrospective study analyzed records of 103 patients (52, 50.5%, men, and 51, 49.5%, women) diagnosed with submandibular infections. The mean (SD) age of patients was 39.3 (12.6) years and ranged from 18 to 73 years. Most patients were identified as White (76, 73.7%), while the remaining patients were identified as Black (27, 26.2%). Of the 103 patients, 37 (36.2%) had the following comorbidities: hypertension (18, 17.4%), diabetes (11, 10.6%), cancer (5, 4.9%), and renal disease (3, 2.9%). The submandibular space was the most commonly involved anatomic space in both single and multiple space infections.

Outcomes for the anatomical extent of infection are reported in Table 1. Of the 103 patients, the majority (93, 90.3%) of patients had multiple space infections, and 10 (9.7%) had single space infections. Of those with multiple infections, the majority (52/103, 50.5%) had a sublingual space infection, but the masticator space (46/103, 44.7%) and submental space (45/103, 43.7%) were also common. Of those with a single infection, the majority (9/103, 8.7%) had an infection in the retromolar trigone region or the carotid space. A review of CT images in the 103 patients indicated that 61 (59.2%) had an abscess, and 42 (40.8%) had cellulitis. More infections (54/103, 52.4%) were found on the left side. The most common complications of infection were submandibular gland enlargement (29/103, 28.2%) and airway obstruction (20/103, 19.4%).

**Table 1:** Anatomical extent of infections in patients (N = 103) with submandibular space infections.

Facial space location of infection	Cases, *n* (%)
Multiple space infections	93 (90.3)
Sublingual space	52 (50.5)
Masticator space	46 (44.7)
Submental space	45 (43.7)
Parapharyngeal space	33 (32.0)
Buccal space	18 (17.5)
Single submandibular infection	10 (9.7)
Retromolar trigone region	9 (8.7)
Carotid space	9 (8.7)
Parotid space	8 (7.8)
Mediastinum	7 (6.8)
Retropharyngeal space	6 (5.8)
Danger space	1 (1.0)

When evaluating the effect of age on infection, the majority of patients with facial infections were aged 31-40 years (32/103, 31.1%) or 21-30 years (27/103, 26.2%) (Table 2). When comparing the extent of infection with hospital LOS, those with airway narrowing had longer mean (SD) hospital LOS (9.3 (9.1) days, *P* = 0.04) (Table 3). The normality assumption was violated for the comparison of LOS between airway narrowing groups. A Mann-Whitney U test yielded results consistent with the original test and remained statistically significant (*U *= 1508, *P* < 0.001). To evaluate the influence of an extreme LOS value, an additional sensitivity analysis was performed excluding this observation. Both the t-test and Mann-Whitney U test remained statistically significant after exclusion of the extreme value (both *P* < 0.01), indicating that the findings were robust to the analytical approach and the presence of the extreme observation. When comparing a second operation with the anatomic extent of infection, those with infection in the retromolar trigone region (χ^2^(1) = 5.3, *P* = 0.02) and submental space (χ^2^(1) = 4.2, *P* = 0.04) were more likely to have a second operation (Table 4).

**Table 2:** Comparison of age range of patients (N = 103) with submandibular space infections.

Age range of patients	Infections, *n* (%)
18-20 years	1 (1.0)
21-30 years	27 (26.2)
31-40 years	32 (31.1)
41-50 years	22 (19.4)
51-60 years	16 (15.5)
61-70 years	7 (6.8)

**Table 3:** Comparison of submandibular space infection type and hospital length of stay (LOS) in patients with submandibular space infections (N = 103). Hospital LOS was measured in days.

Infection type or location	Hospital LOS, mean (SD)	t	*P*-value
Abscess (*n *= 61)	5.8 (3.4)	−0.57	0.57
Bacterial gas (*n *= 34)	5.9 (3.3)	−0.43	0.67
Enlargement of the submandibular gland (*n *= 29)	5.3 (2.9)	0.30	0.76
Airway narrowing (*n *= 20)	9.3 (9.1)	−2.27	0.04
Trismus (*n *= 7)	8.0 (5.1)	−1.34	0.18
Enlargement of the parotid gland (*n *= 6)	13.8 (15.8)	−1.36	0.23

**Table 4:** Comparison between a second operation and the anatomic extent of infection in patients with submandibular space infections (N = 103).

Anatomic extent of infection	Second operation, *n* (%)	*χ*^2^	*P-v*alue
Retromolar trigone region (*n* = 9)	3 (33.0)	5.3	.02
Submental space (*n* = 45)	8 (17.8)	4.2	.04
Sublingual space (*n* = 52)	8 (15.3)	2.4	.12
Parotid space (*n* = 8)	2 (25.0)	1.9	.17
Masticator space (*n* = 46)	7 (15.2)	1.8	.18
Buccal space (*n* = 18)	3 (16.6)	0.8	.37

## Discussion

In this retrospective study, we evaluated the clinical variables associated with the severity of submandibular space infections and their treatment outcomes. Because the spread of submandibular space infection to other areas in the head and neck region may result in serious consequences for patients, we used CT scans to determine whether treatment outcomes, such as hospital LOS or a second operation, could be used as predictors of these outcomes. Overall, our results indicated that most patients had anatomical spread of infection to adjacent facial spaces, which increased their LOS. Further, surgical interventions were more common for patients with abscess formation than cellulitis. However, some patients had delayed recovery from the submandibular infection and required a second operation to drain the abscess. Although the extent of infection was the same between the age groups examined, a slightly higher incidence was seen in the group aged 21-40 years. As indicated by the CT images, the submandibular space was the primary location of infections. Infections in the sublingual, masticator, and submental spaces were commonly found around the mandible, and infections in the parapharyngeal space were commonly along the neck. Most patients had multiple space infections, which was consistent with studies that reported 93% of odontogenic infections involved multiple spaces [3,6]. Less than 10% of patients in our study had single submandibular space infections. In contrast, a study by Bridgeman et al. [10] reported 53% of 107 infections involved a single space. In general, our results indicated that submandibular space infections usually spread to adjacent spaces. In some cases, they may extend as far as the mediastinal space. Other studies have also reported that infection of the submandibular space typically spreads to other deep neck facial spaces [11,12].

In the current study, the sublingual space was the second most involved in submandibular infections. This finding was likely due to the lack of fascia and accessible communication at the posterior border of the mylohyoid muscle between the submandibular and sublingual spaces [3,13,14]. Narrowing of the airway and mediastinal infections have also been associated with sublingual space infection [15]. Similarly, the submandibular and buccal spaces frequently involve fascial spaces in dental infections [16,17]. Our results supported this frequency of submandibular space involvement, but less than 20% of our patients had buccal space involvement. Patients in the current study were more likely to have sublingual, masticator, submental, or parapharyngeal space involvement than buccal space involvement. Given that the submandibular and sublingual spaces seem to be the most common sites of odontogenic infection, clinicians should closely monitor these areas for early signs of infection and implement timely interventions to prevent complications.

About half of our patients had infections in the submental or masticator spaces. The submental space is anterior to the submandibular space between the two anterior bellies of the digastric muscles. Infection can reach this space through the lower incisor teeth or accessible communication with the submandibular space. Consistent with previous studies [16,18], a majority of our patients with submental and sublingual space infection also had airway narrowing. Further, in addition to submandibular space infection, almost half of the patients had masticatory space involvement and enlargement of the masseter and medial pterygoid muscles. Previous studies [11,19] have reported that odontogenic infections often spread first to the masticator space and then into the submandibular space through the masseter and medial pterygoid muscles. However, our results supported the outcomes of other studies that reported masticator space infections were often accompanied by submandibular involvement [3,20,21]. In those studies [3,20,21], the odontogenic infection spread from the mandibular teeth to the submandibular space to the masticator space rather than from the masticator space to the submandibular space.

Although our results suggested that infection extended directly from the masticator space, it may also have spread directly from the submandibular space to the parapharyngeal space. The fascial barrier between these spaces is thin [13,14], and the findings of our study supported the notion of direct communication between submandibular and parapharyngeal space infections [3,13-22]. However, a tenth of our patients had parapharyngeal space infections without masticator space involvement, and almost a quarter had parapharyngeal and masticator space infections. Of those with parapharyngeal space infections, a few had retropharyngeal abscesses. These findings were supported by Kato et al. [23], who suggested that retropharyngeal space infections arose from the parapharyngeal space. In another study, Branstetter and Weissman [24] reported that patients with retropharyngeal space infections were more at risk of airway compromise. However, those authors also reported that only a quarter of patients with retropharyngeal space infections complained of airway narrowing [24]. Results of our study suggested a much higher co-involvement of the retropharyngeal and parapharyngeal spaces. This finding may have important implications for practice because infections in these deep neck spaces can lead to life-threatening complications, such as airway obstruction and mediastinitis.

Because airway narrowing and displacement are common complications of deep neck infections and sometimes lead to death [25], we included airway narrowing as a surgical complication in the current study. Compromise or restriction of the airway is often induced by inflammation and edema but can also be caused by aspiration of abscess material [24,26]. Almost a fifth of patients in our study had airway narrowing, and this complication was significantly associated with a longer hospital LOS. One explanation for this finding may be related to standard medical practices associated with a patient complaint of airway restriction; this complication usually results in immediate hospital admission since airway restriction can lead to life-threatening complications. For our patients with airway narrowing, one or more of the following spaces were involved: submandibular, sublingual, submental, parapharyngeal, and masticator spaces. Similar findings were reported by Zhang et al. [27], who also recommended close monitoring of patients to prevent airway obstruction caused by infection in facial spaces, since those infections may require an emergency endotracheal intubation or tracheotomy as a result of respiratory distress.

In the current study, we also evaluated the likelihood of patients having a second operation. Our analyses suggested that a second operation was significantly associated with retromolar trigone and submental space infections. Because the submental space lies in the midline of the neck, the infection may spread more quickly to the opposite side, complicating unilateral drainage of the infection. Another possible explanation for this finding may be related to the submental space. Involvement of that space may lead to bilateral involvement of the sublingual and submandibular spaces, indicative of a serious condition known as Ludwig angina. As suggested by Lypka and Hammoudeh [28], a better understanding of the anatomy and relationships of the facial spaces of the head and neck region may provide valuable information about the spread of submandibular infection and its surgical management. For example, Kato et al. [23] reported that odontogenic infections spread to the parapharyngeal and retropharyngeal spaces from the pterygomandibular space and through to the submandibular space. Based on our study results, it seems likely that the spread of infection from the retromolar trigone to the pterygomandibular space leads to multiple abscesses that can make clinical management of the infection difficult.

In previous studies of odontogenic infections [6,29,30], the following clinical variables were used as predictors for hospital LOS: certain medical diseases, temperature, lower face infection, admission white blood cell count, location of infection, time spent in the operating room, and infection of non-third molar posterior teeth. In our study, we found that clinical variables related to facial spaces, infection characteristics, complications, and demographic characteristics may also predict hospital LOS.

Our study did not find a significant correlation between immunocompromised patients who had cancer or diabetes patients who were under control and hospital LOS. This result was inconsistent with the study by Ylijoki et al. [8], who reported that lengthy hospital stays occurred because of deep facial infections associated with immunosuppressive conditions. This difference may be due to the small number of patients in our study. Small sample size may also explain why no significant correlations were found for the other systemic diseases of hypertension and renal disease.

The current study had several limitations. The age range of patients included in our retrospective study was extremely broad (i.e., 18-73 years old), which likely affected our ability to evaluate age-based effects of submandibular space infections and treatment outcomes. Similarly, the majority of patients were White, which likely limited the generalizability of our findings to the general population. Another limitation was related to our data collection methods, such that some methods may have been inappropriate for study variables. Additionally, because the study was conducted from 2011-2014, we used the ICD-9 codes instead of the more recent ICD-10 codes, which may affect the reproducibility of our results and limit direct comparisons with more recent studies. Another limitation of the study was the limited representation of racial groups in the patient records. Only White and Black patients were represented, which may be related to the retrospective nature of the study, the sample size, and the electronic medical records. Future studies should be conducted to address these limitations, and a prospective study may be useful to confirm our findings.

## Conclusions

Because odontogenic infections can become life-threatening if they spread to deep facial tissues in the head and neck, this study evaluated clinical outcomes associated with the extent of submandibular space infections and their treatment outcomes. Our results indicated that the submandibular space was the most common location for odontogenic infection. Spreading of infection to the retromolar and submental facial spaces was identified as the variable most strongly associated with increased extent of infection. Because of the complex nature of the anatomy of this space, the spread of the infection can be unpredictable. Therefore, it is necessary to better understand the anatomic location of odontogenic infections and their involvement with surrounding structures. Such information could improve the diagnosis, management, and treatment of head and neck infections and, ultimately, lead to better clinical outcomes for patients.
